# Simulation of Fish Acute Toxicity of Pharmaceuticals Using Simplified Molecular Input Line Entry System (SMILES) Notation as a Representation of Molecular Structure

**DOI:** 10.3390/ijms26199348

**Published:** 2025-09-24

**Authors:** Alla P. Toropova, Andrey A. Toropov, Erika Colombo, Edoardo Luca Viganò, Anna Lombardo, Alessandra Roncaglioni, Emilio Benfenati

**Affiliations:** Laboratory of Environmental Chemistry and Toxicology, Department of Environmental Health Science, Istituto di Ricerche Farmacologiche Mario Negri IRCCS, Via Mario Negri 2, 20156 Milan, Italy; andrey.toropov@marionegri.it (A.A.T.); erika.colombo@marionegri.it (E.C.); edoardo.vigano@marionegri.it (E.L.V.); anna.lombardo@marionegri.it (A.L.); alessandra.roncaglioni@marionegri.it (A.R.); emilio.benfenati@marionegri.it (E.B.)

**Keywords:** fish acute toxicity, pharmaceuticals, quantitative structure–activity relationship (QSAR), Monte Carlo method, SMILES, index of ideality of correlation (IIC)

## Abstract

The practice of using optimal descriptors has been applied for more than twenty years to develop in silico models. In the present study, a series of in silico models was built to predict the acute fish toxicity of pharmaceuticals using optimal descriptors. The SMILES format was used to represent the chemical structure. The data were split into five training and validation sets. The obtained model for fish toxicity yielded a determination coefficient of 0.67 for the external validation set, representing an acceptable quality, considering the complexity of the pharmaceuticals given their molecular structure and specific biological activity. This study is useful for assessing the acute fish toxicity of pharmaceuticals and, in general terms, as an approach to building models for complex biological endpoints.

## 1. Introduction

The construction and use of QSAR models is a convenient approach for studying and predicting the values of different types of endpoints [1,2,3]. Acute toxicity to fish is important for evaluating the impact of chemical substances on aquatic ecosystems [4,5]. The health of fish is directly linked to the quality of their food and other sources of contamination; furthermore, pollutants accumulated in fish can be transferred to humans consuming contaminated fish, and there are, in fact, well-known cases of these events. Thus, regulators have applied threshold values for contaminants in fish [6,7].

QSAR models for fish and aquatic species have been developed, exploring different topics. The mechanism of action is an important aspect of the simulation of toxicity to fish [8,9]. The comparison of toxicity to fish versus other species is another perspective that provides benefits from the information available from other aquatic organisms [9,10]. The comparison of the effects on aquatic organisms of different ages is another useful approach for developing QSAR models [11]. A comparative study of the impact of pesticides on various ecological systems, as well as the impact of pesticides on the organisms living within them, has been evaluated [7,12,13]. Naturally, these complex problems necessitate the development of new mathematical techniques and algorithms [14,15,16]. In addition, food chains should also be considered from the point of view of developing and improving QSAR analysis [17,18]. Recently, the list of traditional substances has been supplemented by nanomaterials; this requires new solutions in terms of strategy and tactics of modeling processes [4,19]. Models on industrial chemicals are more easily developed due to the availability of high-quality, curated databases containing a wide range of chemicals. However, some studies have explored other substances [20,21]. Numerous studies on the toxicity of pharmaceuticals to fish have been reported, and a review on these models has been published [22]. Pharmaceuticals offer a particular case of environmental pollution affecting fish. These substances are designed to have specific activities with very effective biological mechanisms. Their structure is typically more complex than classical industrial chemicals, and their effects may occur at quite low concentrations. All these factors complicate any investigation of the ecotoxicological impact of pharmaceuticals. For instance, endocrine disruption has been reported due to pharmaceuticals in effluents from wastewater treatment plants, leading to a feminization of the species [23]. Pharmaceuticals can alter neurotransmitter levels in fish [24]. The effects on fish may relate to changes in their behavior, and this may alter their ability to survive and respond to risks and predators [25]. A list of various effects on fish has been published [22], and it is clear that the specific and very diverse types of activity associated with pharmaceuticals pose a challenge from an ecotoxicological point of view, and with therapeutic use, the biological activity may conversely have adverse effects in fish. For this reason, the European Medicinals Agency has published a review and advanced guideline for environmental risk assessment, aiming to minimize the adverse effects of pharmaceuticals [26].

The experimental measurement of the toxicity of all pharmaceuticals in fish would require decades and large numbers of animals for in vivo experiments, with very high costs and ethical concerns [27]. For this reason, as part of the PREMIER—Prioritisation and risk evaluation of medicines in the environment. Innovative Medicines Initiative 2 Project (available online: https://imi-premier.eu, accessed on 7 September 2025), we explored the possibility of developing models of fish acute toxicity using in silico methods. A mathematical function is built, typically using molecular descriptors to codify chemical information assumed to be linked to the endpoint. Numerous models are available to predict fish acute toxicity, such as those in the VEGAHUB platform-Istituto di Ricerche Farmacologiche Mario Negri IRCCS (available online: https://www.vegahub.eu, accessed on 7 September 2025). This platform offers seven models to predict fish toxicity values (plus a classifier model). However, when we tested these models on pharmaceutical compounds, their performance was poor. This is due to the peculiarity of the pharmaceuticals. Chemicals may be represented by molecular descriptors or graphs in other cases. In our study, we used a different representation, simply starting from the SMILES [28]. In this case, the algorithms and logic for calculating molecular descriptors differ conceptually from the descriptors calculated from molecular graphs [29,30,31,32,33,34,35,36]. The algorithm used for the model is simpler and does not require the calculation of molecular descriptors. We applied different algorithms to optimize the results, including the IIC [37,38,39]. The main essence of the IIC is that it is simultaneously sensitive to two statistical characteristics: the correlation coefficient and the mean absolute error. Thus, the software identifies the best results by simultaneously considering the optimization performed on these two parameters. IIC was applied in QSAR analysis previously, and it proved to be useful [40,41,42,43,44,45,46].

## 2. Results

**Figure 1:**
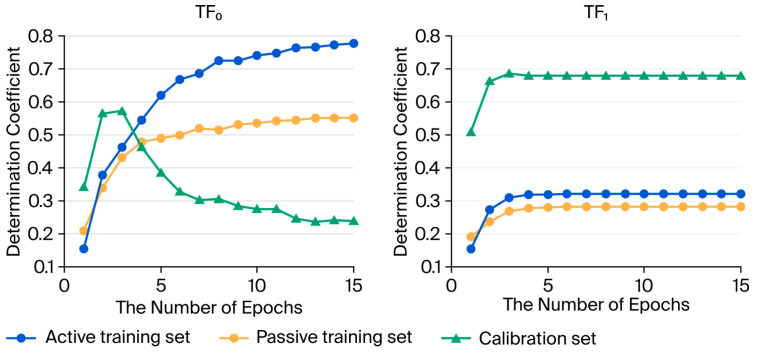
Monte Carlo optimization histories using functions TF_0_ and TF_1_. Special markers indicate the values of the correlation coefficients for the active and passive training sets and the calibration set, spanning 1 to 15 iterations of Monte Carlo optimization. TF_1_ gives stable, high values for the coefficient of determination of the calibration set. TF_0_ leads to an increase in the coefficient of determination for training sets. However, for the calibration set, the maximum of the coefficient of determination is reached, followed by a decrease in its values.

We developed new QSAR models for fish acute toxicity, addressing in particular pharmaceutical substances. We used two equations associated with two target functions. The difference is the use of the IIC parameter [37,38,39]. If we follow the optimization of correlation weights using these two target functions, we can see that they have significantly different optimization outcomes. For TF_0_, overtraining is visible in Figure 1. Specifically, there is an increase in the determination coefficient of the training sets, and a decrease in the case of the calibration set. Conversely, for TF_1_, no overtraining is observed, and this is useful. We see that the determination coefficient for the training sets is noticeably lower than that for TF_0_. These two sets are used in the initial phase of the model development. The model is complete when it is optimized, and this is performed using the calibration set. Thus, in the case of the model using the TF_0_, we observe what could be assumed to be a good model based on the initial results on the active training and passive training. However, the model provides poor results when it is applied to the calibration set. The model is too adapted to the initial, small set (approximately 25% of the substances), which is the active training set. Further evolution and checks, as performed with the passive training set, show that the model based on the active training was poor and could not be improved. Finally, in the last phase of the modeling process, using the calibration set, the model cannot be optimized. Only in its first evolution, with a very small number of epochs, the results on the calibration set were above the value of 0.5. When the epochs are increased, the model evolves too much, trying to replicate the values of the active training set, without the possibility of generalizing the model.

The opposite situation is noted for the TF_1_. The algorithm improves when the initial parameters based on the active and passive training sets evolve. An increase in the performance obtained through final optimization of the model, which is achieved with the use of the calibration set data and optimization of the model parameters, indicates that the process of model building is proceeding correctly.

**Figure 2:**
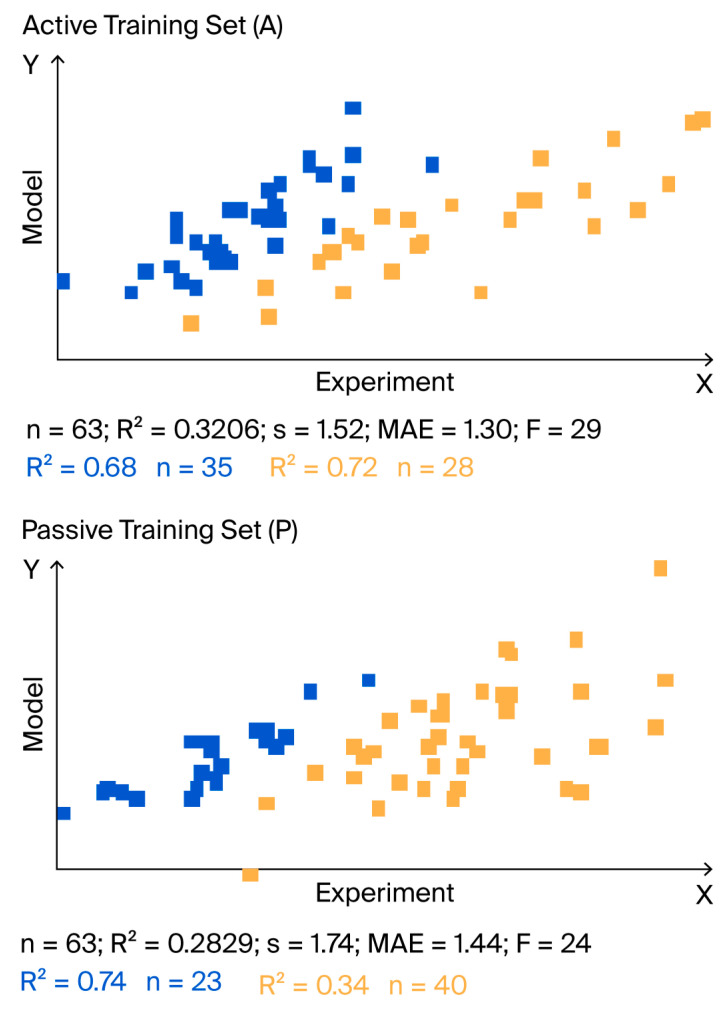
Clustering of points in the ‘experiment–model’ coordinate space observed in split 1 during Monte Carlo optimization using target function TF1. Blue color shows points where the experimental value of the endpoint is less than the predicted value. Yellow color shows points where the experimental endpoint value is greater than or equal to the predicted value. Of note, the coefficients of determination for the blue and yellow clusters are significantly larger than the total value of the coefficient of determination for the overall active and passive training sets.

Figure 2 shows the IIC accompanied by clustering for QSAR models of acute toxicity in fish (split 1, TF_1_). A similar clustering was observed when IIC was involved in the Monte Carlo optimization for model mutagenicity described in the literature [39]. This explains why the determination coefficients for active and passive training sets are moderate or even low.

Table 1 shows the statistical parameters of the models TF_0_ and TF_1_ constructed using CORAL software-2024 (available online: http://www.insilico.eu/coral, accessed on 7 September 2025).

Given that the values of the active and passive training sets represent the values observed in the initial phases of the model development, they are reported but are used only internally. The values obtained with the calibration set (which is the final model) represent the statistical parameters for the substances used to develop the model. A model is useful if it provides good results when tested on substances never used to build the model (which are the chemicals in the validation set). Thus, when reading Table 1, the most relevant values are those pertaining to the calibration and, in particular, the validation sets. The model derived using TF_0_ and the optimal descriptor of the correlation weights of SMILES attributes DCW(1,3) did not produce good results. The average determination coefficient for the validation set is 0.56 (its dispersion is 0.07). In case the target function TF_1_ (DCW(1,15)), the average determination coefficient for the validation set is 0.67 (its dispersion is 0.04). Thus, the Monte Carlo optimization with the target function TF_1_ has better predictive potential than that with the target function TF_0_.

The described computational experiments show that the use of TF_1_ is effective in achieving an increase in the coefficients of determination for the external validation sets. This is an advantage of the approach, which uses the index of ideality of correlation. The latter has found application for the simulation of several quite diverse endpoints [40,41,42,43,44,45,46].

### 2.1. Search for Outliers

The applicability domain for the models under consideration is determined by the so-called statistical defects, which are used for the search for outliers. Statistical defects enable us to judge how closely a molecular structure compares with other structures in the model. A small statistical defect indicates the substance is probably not very different from other structures and therefore should not be an outlier [39]. According to this criterion, the best model (optimization with TF_1_, split 1) has a total of 31 outliers, nine of which are in the external validation set.

### 2.2. Mechanistic Interpretation

The model can be mechanistically interpreted by comparing the results with different splits to verify that the features associated with the assumed mechanism are consistently derived from different splits [39]. Those SMILES attributes (defined in Section 2.3) that exhibited a positive correlation weight across all optimization trials are considered promoters of an increased endpoint. Similarly, those with a negative correlation weight across all optimization trials are considered promoters of lower values of the endpoint. Table 2 gives the results of three trials of the optimization with TF_1_ for split 1. The majority of SMILES attributes can be interpreted according to the SMILES concept [28]. These SMILES attributes are in many cases single atoms (Cl, O, S, N), a number of rings (represented by digits 1, 2, etc.), one atom in a certain condition (linked to a ring or atom with a particular bond), or two atoms (carbon-carbon where both are aliphatic or carbon-carbon where one is aromatic and one is aliphatic).

Among the molecular components identified by the model, one can recognize some features often associated with higher fish acute toxicity. Specifically, these molecules have a positive CW. For instance, chlorine is identified by the model as a simple atom within a branched structure (indicated by the parentheses in the SMILES notation). As an example, hexachlorophene (CAS is 70-30-4, SMILES is Oc1c(Cl)cc(Cl)c(Cl)c1Cc1c(O)c(Cl)cc(Cl)c1Cl) characterized by pLC50 = 1.68. In the case of sulfur, tetraethylthiuram disulphide (CAS is =97-77-8, SMILES is CCN(CC)C(=S)SSC(=S)N(CC)CC) characterized by pLC50 = 0.49.

The presence of multiple rings, both aliphatic and aromatic (as in the case of three rings), is associated with an increased toxicity; this can be explained by the higher lipophilicity of these large substances with many carbons. Indeed, many of the common QSAR models for fish acute toxicity use logP as one of the descriptors, as is the case of the fathead minnow LC50 96h model developed by the US EPA-U.S. Environmental Protection Agency (available online: https://www.epa.gov/, accessed on 7 September 2025) and also implemented in VEGAHUB (available online: www.vegahub.eu, accessed on 7 September 2025). For instance, the substance with CAS is 97-23-4 and SMILES is Oc1ccc(Cl)cc1Cc1cc(Cl)ccc1O has pLC50 = 0.510 [47].

The model identifies the relationships between toxicity and the number of rings in particularly in the case of two or three rings, but not for a higher number of rings. This can be due to multiple reasons. The number of substances with four or more rings is smaller, and this may have an impact. Furthermore, if the molecule becomes too large, its water solubility is reduced. Thus, the substance, even if it is bioaccumulative and thus potentially toxic in principle, is not sufficiently water-soluble [48]. In our case, we calculated logP with the VEGAHUB (available online: www.vegahub.eu, accessed on 7 September 2025). and considered a substance with pLC50 values above or below 0 (taken as a value for reference for toxicity). For substances with one ring, the average logP value is 1.3 for substances with pLC50 below 0, and 2.6 for substances with pLC50 above 0. For substances with two or three rings, the average logP is 2.0 in the first case, and 5.1 in the second case; thus, there is a large difference in logP between toxic and non-toxic substances. For substances with four or more rings, there is no difference in toxicity, related to logP values, since the average values are 2.1 and 2.5 for toxic and not-toxic substances, respectively. Conversely, the SMILES attributes with a negative CW are associated with lower toxicity. In this category, there are substances containing oxygen and nitrogen, which increase the polarity of the substance, lowering its bioconcentration.

### 2.3. Comparison of the Results with Other QSAR Models

Table 3 compares the model developed here with those described in the literature. The model suggested here performs quite well compared with models from the literature.

As discussed in the Introduction, there is a wide range of studies on different types of substances, and it is not our intention to provide a comprehensive review. Table 3 provides a representative picture of the different cases that may be found in the literature. The first example, with 65 substances, refers to pesticides. This type of substance is somehow similar to pharmaceuticals, considering the complexity of the chemical structures and the biological mechanisms. Thus, the statistical parameters obtained in this study may be compared with those we obtained. The study using 14 substances is an example of a good model. However, this model cannot be directly compared with our model because the study refers to industrial chemicals, which typically have a simpler chemical structure compared to pharmaceuticals. Furthermore, the model is relative to only 14 chemicals; thus, it is a focused, local model. The third example uses 107 substances. The dataset is larger than the previous datasets, but the substances are primarily homogenous substances. Thus, our study on pharmaceuticals may be preferably compared with the situation of pesticides. Indeed, the values are quite similar. In both cases, we have complex chemical structures containing heterogeneous substances that are not limited to a single family and demonstrate activity through multiple pathways. Thus, this is probably one of the most challenging situations. Other modeling approaches for pharmaceuticals can likely be applied, achieving similar results. Our approach, in contrast to others one gives the possibility to compare and weigh various molecular fragments by stochastic processes. In addition, the algorithm is based on the Monte Carlo approach. The strategy to split the initial set into different subsets is more complex, but does not involve any particular effort from a calculation point of view. In [20,27,49], the SMILES representation of molecular structures was used. Attempts were made to represent toxicity models for these datasets using the technique discussed in this section. That is, partitions were constructed into the four subsets, followed by Monte Carlo optimization. Table 3 compares the original models and those obtained using the methodology described here. This showed that the described Monte Carlo models have predictive potential (average for five different splits) comparable to the original models (Table 3 and Table S2).

## 3. Discussion

A peculiar aspect of this work is the use of the index of ideality correlation [37,38,39]. The main objective of this index is to be sensitive to both the most important statistical criteria: the magnitude of the correlation coefficient and the magnitude of RMSE (or MAE). Most of the phenomena in the natural sciences are complex.

At the beginning of QSPR/QSAR theory development, establishing correlations between descriptors derived from the molecular structure and endpoints was considered the primary goal of the research. Further QSPR/QSAR studies have shown that the real predictive potential of the model for the training set and the correlations outside the training set occasionally (or even usually) exhibit significant discrepancies. Graphically, this can be observed through the plot of the coordinates “observed-predicted” values of the endpoint, where asymmetry may be observed. In particular, points may lie on the diagonal for substances used to build the model, indicating a good correlation. The IIC is a criterion for assessing the predictive potential of QSPR/QSAR models by assessing the above-mentioned “asymmetry”.

All models start from a partial representation of the complexity of the problem to be addressed. We use a limited number of substances, representative of a limited number of processes. Furthermore, in all modern modeling approaches, the available substances are typically split into training and validation sets, as is common practice. It should be considered that virtually every split of the data into a training set and a validation set generates a model, highlighting the inherent randomness of QSAR models. This is partly natural and obvious, since variations in the activities of substances (even in homologous series) are random in nature. To reduce the bias and to move towards stability in conclusions, several splits into a training set and a validation set should be studied. If the number of substances available is small, high variability is expected with different splits.

The strategy of constructing models using the IIC is specific. Although the calculation of correlation weights is performed on the active and passive training sets, the assessment of the quality of the model is partially performed on the statistical characteristics related to the calibration set. Given the strategy described, the calculation of the correlation weights is self-organized.

The hypothesis of the existence of molecules with average behavior and molecules with atypical behavior [35] is quite realistic. In fact, among molecular behaviors, there is room for both docile molecules functioning in a standard range and for atypical molecules exhibiting special behaviors that render them outliers in optimized models.

IIC responds to the presence of atypical molecules and their behavior. As a result of this sensitivity, the superposition of points in the “experiment-calculation” coordinates splits into two clusters. When assessed based on the overall correlation coefficient across both clusters, the value of the correlation coefficient is small or even close to zero; thus, the statistical parameters of the active and passive training sets may be apparently poor. However, when the two clusters are considered separately, the correlation coefficients can be good (see Figure 2, with an R^2^ of 0.32 for the total active training set, whereas separate R^2^ values are 0.72 and 0.64).

The specified configuration of two correlations resulting from the use of IIC with Monte Carlo optimization may be a compromise that prevents overtraining. This method stabilizes the correlation structure, preserving the predictive potential when the model loses consistency for training samples, but gains consistency for the calibration set.

The internal difference between the optimization of the objective function TF_0_ and the objective function TF_1_ is shown in Figure 1. It is evident that without using IIC, optimization using the Monte Carlo method leads to a continuous increase in correlation of the values for the active and passive training sets. Conversely, for the calibration set, the process proceeds to a certain maximum, after which there is a decrease in correlation.

So far, we have discussed the results for the different subsets of substances involved in the model development. An important question arises: do the aforementioned optimization strategies improve the statistical quality of the model for the external validation set? Unfortunately, there is virtually no rigorous mathematical evidence that suggests an improvement in the statistical characteristics of models when applied to the validation set. However, there are models for various endpoints where such an improvement was observed [37,38,39]. In the present study, a comparison of the determination coefficients and RMSE values observed with the use of the target functions TF_0_ and TF_1_ allows for the identification of differences between these models.

**Figure 3:**
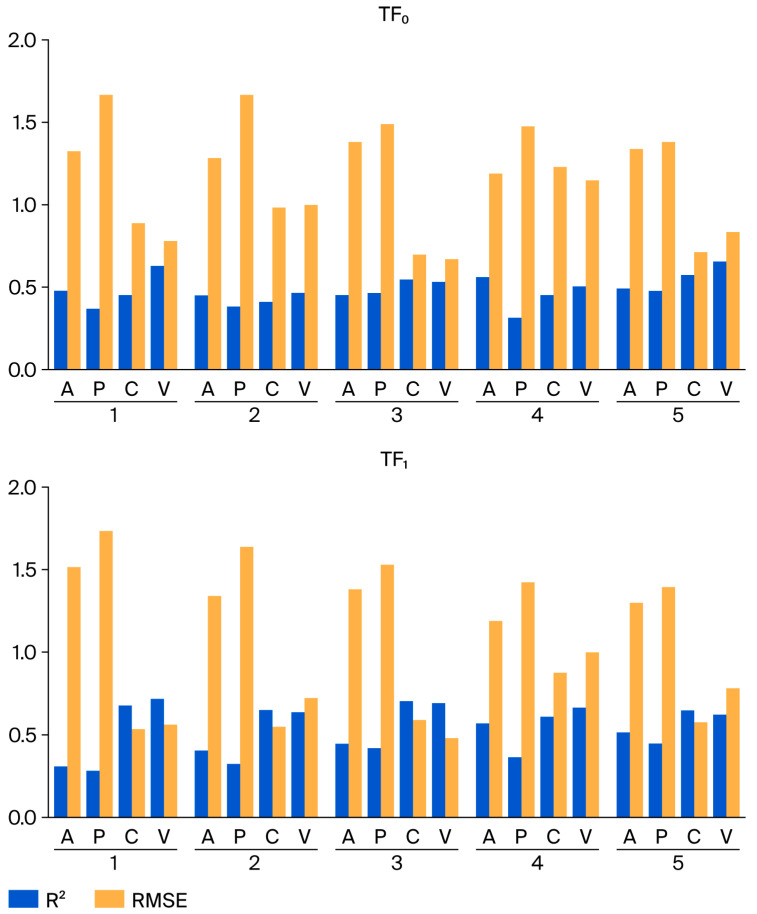
Comparison of determination coefficients and RMSE observed for TF_0_ optimization and TF_1_ optimization.

First, the numerical values of the coefficients of determination in the case of TF_0_ optimization appear disorganized, whereas under TF_1_ optimization, a clear ranking by values is observed (Figure 3). For the calibration and validation sets, the values of the coefficients of determination are greater than those for the active and passive training sets. Second, the RMSE values are also disordered under optimization using TF_0_ and explicitly ordered for optimization using TF_1_. Third, the average value of the determination coefficient for the validation set in the case of optimization using the objective function TF_0_ is 0.56 ± 0.07. However, for optimization using TF_1_, the average value of the determination coefficient for the validation set is 0.67 ± 0.04. It should be noted that the dispersion of coefficient of determination values on validation sets is an informative parameter. Naturally, the lower the mentioned dispersion is, the higher the confidence in the model.

In summary, the approach used here is a compromise between mathematical rigor and stochastic reality. In fact, the QSAR model is a random event, and the splitting of the data into training and validation sets is the basis of this random model [50,51,52,53,54]. It is possible to build a very large number of similar models using various splits. In our case, five random splits were considered. This approach will most likely useful in other cases because the Monte Carlo technique is generally quite universal [55,56,57,58,59].

Thus, the problem of the toxic effects of pharmaceutic on fish is an actual problem [60,61,62].

## 4. Materials and Methods

### 4.1. Database

The toxicity decimal logarithm values (pLC50, in mg/L) for 251 chemicals were retrieved from the following sources: (1) the PREMIER consortium, which includes shared data from the IMI-iPiE project—Innovative Medicines Initiative-the identification of the potential environmental risks of existing and new active pharmaceutical ingredients https://www.ihi.europa.eu/projects-results/project-factsheets/ipie, accessed on 7 September 2025), EPAR-European public assessment report (https://www.ema.europa.eu/en/medicines/what-we-publish-medicines-when/european-public-assessment-reports-background-context#topics, accessed on 7 September 2025), US Environmental Protection Agency- ECOTOX Knowledgebase (available online: https://cfpub.epa.gov/ecotox/index.cfm, accessed on 7 September 2025), and EFPIA—European Federation of Pharmaceutical Industries and Associations (https://www.efpia.eu, accessed on 7 September 2025), and (2) the literature [20,21,48]. Most of these data are derived from studies conducted according to official protocols. When SMILES notions were not provided, we retrieved them automatically using in-house Chemical-Resolver software (available online: https://github.com/EdoardoVigano/Chemical-Resolver, accessed on 7 September 2025). The retrieved SMILES notions were then canonized using in-house the-Chemical-Smiler software (available online: https://github.com/davideLuciani165/The-Chemical-Smiler, accessed on 7 September 2025) to make them suitable for in silico modeling. For duplicates with different experimental values, we applied the following data prioritization:

(1) Data provided by PREMIER were maintained;
(2) The second choice was data provided by EFPIA;
(3) In case of duplications from the literature, we selected data that included information about the species [63,64,65].
(4) In case of duplicates in PREMIER data, we considered the more generic stereochemistry structure because our in silico model cannot deal with this chemical information.

The LC50 was used as the endpoint for modeling, which represents 50% lethality in tested organisms. These chemicals were randomly distributed into the active training (25%), passive training (25%), calibration (25%), and validation sets (25%). Each of these sets has a specific task [37]. In our approach, most of the available data and related information are used for the model’s preparatory steps (passive training and calibration sets account for 50%), whereas only 25% is used for the final model construction (active training) [37].

(i) The active training set is the set used to initially build the model, i.e., compounds of this set are used to build the predictive model.
(ii) The passive training set is the inspector of the model, i.e., compounds of this set are used to assess whether the model is satisfactory for substances that are absent in the active training set.
(iii) The task of the calibration set is to detect the start of the overtraining using an increased number of epochs.
(iv) The validation set is used for the final validation of the predictive potential of the model.

### 4.2. Simulation

The models considered here are defined in Equation (1):

(1) $$p{LC}_{50}=C_{0}+C_{1}\times DCW(T,N)$$

The DCW is the descriptor of the correlation weights of SMILES attributes, whereas $C_{0}$ and $C_{1}$ are the regression coefficients of the equation. In particular, SMILES attributes are (i) SMILES atoms, i.e., fragments of SMILES that are one symbol or a group of symbols that cannot be considered separately (‘Cl’, %11, etc.); and (ii) pairs of SMILES atoms that are neighbors in the SMILES string.

### 4.3. Monte Carlo Optimization

Correlation weights are calculated using stochastic Monte Carlo optimization with parameters T and N. T is the threshold, i.e., the minimum number of occurrences of an attribute in chemicals of the active training set at which the attribute is considered active (i.e., non-rare: rare attributes are indicated by correlation weights equal to zero). N is the number of epochs of the stochastic optimization by the Monte Carlo technique (CORAL software, http://www.insilico.eu/coral, accessed on 7 September 2025).

**Figure 4:**
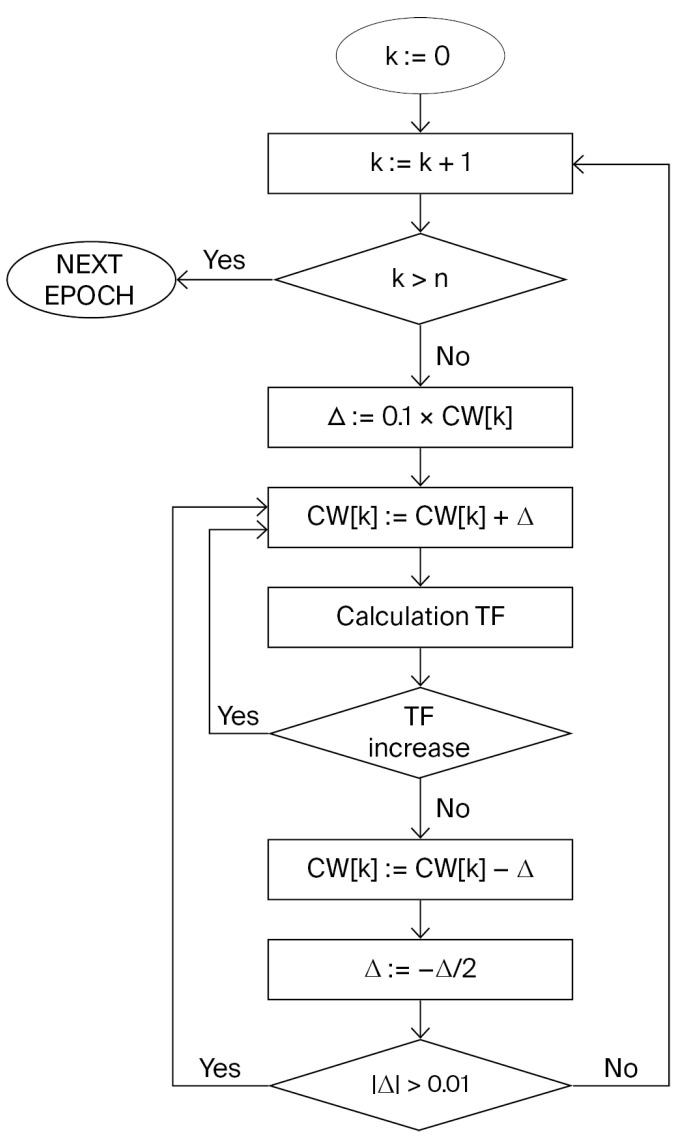
The Monte Carlo optimization workflow. The index k represents the position in the sequence of SMILES attribute value updates that contribute to improving the target function during Monte Carlo optimization.

The version of Monte Carlo optimization used begins with a random vector, the components of which are the correlation weights of various SMILES attributes that have sufficient representation (appearing at least T times) in the active training set. Each iteration (epoch) is a process of improving the correlation weights of all frequent SMILES attributes. The sequence of SMILES attribute selection is random. Figure 4 shows the general scheme of the Monte Carlo optimization.

### 4.4. Descriptor

The descriptor of the correlation weights is the simple sum of the correlation weights of active SMILES attributes, as noted in Equation (2):

(2) $$DCW\left(T,N\right)=\sum CW\left(S_{k}\right)+\sum CW({SS}_{k})$$

S_k_ is an SMILES atom, and SS_k_ is a pair of SMILES atoms that are neighbors in the SMILES string. CW(S_k_) and CW(SS_k_) are their correlation weights obtained in the Monte Carlo optimization of the selected target function.

### 4.5. The Monte Carlo Optimization

The optimization applied here can be performed with different target functions. Two target functions, namely, TF_0_ and TF_1_, were studied here (Figure 1).

(3) $${TF}_{0}=R_{A}^{2}+R_{p}^{2}+\left|R_{A}^{2}-R_{P}^{2}\right|\times 0.1$$

(4) $${TF}_{1}=R_{A}^{2}+R_{p}^{2}+\left|R_{A}^{2}-R_{P}^{2}\right|\times 0.1+0.25\times IIC$$

R^2^_A_ and R^2^_P_ are determination coefficients between the descriptor calculated using Equation (2) and pLC50 for active and passive training sets, respectively. The IIC is the index of ideality of correlation [37,38,39].

### 4.6. Applicability Domain

The applicability domain for the described model, calculated with Equation (1), defines the so-called statistical defects of SMILES attributes [30]. These defects can be calculated as:

(5) $$d_{k}=\frac{\left|{P(A}_{k})-P^{\prime }(A_{k})\right|}{N\left(A_{k}\right)+N^{\prime }(A_{k})}+\frac{\left|{P(A}_{k})-P^{\prime \prime }(A_{k})\right|}{N\left(A_{k}\right)+N^{\prime \prime }(A_{k})}+\frac{\left|P^{\prime }(A_{k})-P^{\prime \prime }(A_{k})\right|}{N^{\prime }\left(A_{k}\right)+N^{\prime \prime }(A_{k})}$$

where P(A_k_), P′(A_k_) P″(A_k_) are the probability of A_k_ in the active training set, passive training set, and calibration set, respectively; N(A_k_), N′(A_k_), and N″(A_k_) are frequencies of A_k_ in the active training set, passive training set, and calibration set, respectively. The statistical SMILES-defects (D_j_) are calculated as:

(6) $$D_{j}=\sum_{k=1}^{NA}d_{k}$$

where NA is the number of non-blocked SMILES attributes in the SMILES.

A SMILES falls in the domain of applicability if

(7) $$Dj<2\;*\;\bar{D}$$

## 5. Conclusions

The computer-based experiments described indicate that optimizing the correlation weights of non-rare (active) SMILES attributes yields satisfactory models for predicting the fish acute toxicity of pharmaceuticals. When the number of optimization epochs approaches infinity, overtraining occurs. This results in high statistical performance for the training sets. However, this is accompanied by decreases in the statistical quality of the calibration and validation sets. Modifying the objective function to include the index of ideality of correlation (IIC) has a positive impact on the Monte Carlo optimization process, improving the statistical quality of the model for the calibration set, but to the detriment of the statistical quality of the training sets. This was accompanied by unexpected separation into two correlation clusters for the training sets. It is important that the scheme described for constructing models corresponds to the known OECD principles. Specifically, its applicability domain is defined, and it provides opportunities for mechanistic interpretation of the resulting models.

## Figures and Tables

**Table 1:** The statistical parameters of models observed in the case of optimization with target function TF_0_ with DCW(1,3) and TF_1_ with DCW(1,15).

Target Function	Split	Set *	*n*	R^2^	CCC	IIC	Q^2^	RMSE	MAE	F
**TF_0_**	1	A	63	0.4791	0.6479	0.5904	0.4405	1.33	1.13	56
		P	63	0.3648	0.5118	0.3703	0.3295	1.67	1.35	35
		C	65	0.4507	0.6380	0.5642	0.3651	0.889	0.611	52
		V	60	0.6360	-	-	-	0.78	0.61	-
	2	A	62	0.4473	0.6182	0.5878	0.4126	1.29	1.10	49
		P	59	0.3894	0.5497	0.2845	0.3546	1.67	1.33	36
		C	63	0.4161	0.5352	0.4373	0.3736	0.996	0.779	43
		V	67	0.4696	-	-	-	1.01	0.73	-
	3	A	66	0.4589	0.6291	0.5310	0.4243	1.39	1.18	54
		P	60	0.4700	0.4753	0.3848	0.4389	1.50	1.26	51
		C	59	0.5530	0.7404	0.7424	0.5196	0.699	0.530	71
		V	66	0.5378	-	-	-	0.68	0.52	-
	4	A	66	0.5687	0.7251	0.6285	0.5453	1.20	0.968	84
		P	64	0.3214	0.5503	0.4334	0.2719	1.48	1.19	29
		C	62	0.4549	0.6063	0.5552	0.4100	1.23	0.922	50
		V	59	0.5075	-	-	-	1.15	0.88	-
	5	A	60	0.4992	0.6659	0.6182	0.4708	1.34	1.11	58
		P	66	0.4753	0.6076	0.5263	0.4412	1.38	1.17	58
		C	61	0.5726	0.7344	0.6651	0.5377	0.716	0.527	79
		V	64	0.6604	-	-	-	0.84	0.64	-
**TF_1_**	1	A **	**63**	**0.3206**	**0.4856**	**0.4530**	**0.2680**	**1.52**	**1.30**	**29**
		**P**	**63**	**0.2829**	**0.4209**	**0.3449**	**0.2438**	**1.74**	**1.44**	**24**
		**C**	**65**	**0.6803**	**0.8225**	**0.8248**	**0.6608**	**0.541**	**0.418**	**134**
		**V**	**60**	**0.7189**	**-**	**-**	**-**	**0.57**	**0.43**	**-**
	2	A	62	0.4053	0.5768	0.5968	0.3562	1.34	1.16	41
		P	59	0.3246	0.4869	0.3736	0.2807	1.65	1.37	27
		C	63	0.6543	0.8026	0.8089	0.6251	0.557	0.456	115
		V	67	0.6404	-	-	-	0.73	0.52	-
	3	A	66	0.4588	0.6290	0.5999	0.4209	1.39	1.24	54
		P	60	0.4267	0.4518	0.3712	0.3911	1.53	1.29	43
		C	59	0.7075	0.8304	0.8410	0.6850	0.596	0.470	138
		V	66	0.7027	-	-	-	0.49	0.41	-
	4	A	66	0.5773	0.7320	0.7151	0.5509	1.19	0.980	87
		P	64	0.3646	0.5932	0.4799	0.3198	1.43	1.13	36
		C	62	0.6193	0.7593	0.7856	0.5905	0.886	0.670	98
		V	59	0.6744	-	-	-	1.00	0.77	-
	5	A	60	0.5218	0.6858	0.5524	0.4953	1.31	1.05	63
		P	66	0.4557	0.5894	0.5492	0.4178	1.40	1.22	54
		C	61	0.6524	0.8032	0.8077	0.6140	0.577	0.427	111
		V	64	0.6276	-	-	-	0.78	0.60	-

* A = active training set; P = passive training set; C = calibration set; V = validation set; R^2^ = determination coefficient; CCC = concordance correlation coefficient; IIC = index of ideality of correlation; Q^2^ = cross-validated R^2^; RMSE = root mean squared error; MAE = mean absolute error; F = Fischer F-ratio. ** The values in bold type indicate the best model represented in Supplementary Materials as Table S1.

**Table 2:** Promoters of an increase or decrease in decimal logarithm of the concentration that is lethal for 50% of the exposed population (pLC50) in mg/L for fish acute toxicity (based on Monte Carlo optimization with TF_1_, split 1).

S_k_ and SS_k_	CWs Probe 1	CWs Probe 2	CWs Probe 3	NA *	NP	NC	Statistical Results
CC	0.5820	0.8181	0.4444	41	43	39	0.0013
cc	0.3720	1.1446	0.7268	37	34	38	0.0009
c1	0.8266	0.8609	0.2485	31	30	40	0.0028
2(	0.3638	0.9570	0.4046	20	8	4	0.0160
C=	0.2293	1.1986	0.6943	20	18	20	0.0011
Cl	1.0875	1.2502	1.0198	12	8	9	0.0044
Cl(	0.9531	0.9448	1.2444	11	6	8	0.0063
cC	0.9585	1.2576	1.3355	11	7	18	0.0092
C3	0.5438	0.0123	0.3713	8	3	5	0.0099
cO	0.7980	0.2225	0.4021	8	9	9	0.0012
S	1.8502	2.1259	2.9819	6	9	2	0.0132
3(	0.3044	1.0361	0.8304	5	4	2	0.0088
O1	0.0996	0.5437	0.1507	5	4	12	0.0115
c3	0.9363	1.2044	0.3981	5	5	2	0.0081
=1	2.8638	3.3565	1.4123	4	2	4	0.0063
O	−0.3447	−0.4275	−0.0051	53	48	56	0.0013
1	−0.2970	−0.5514	−0.1329	47	42	50	0.0015
c(	−0.4694	−0.2529	−0.0718	34	31	34	0.0010
2	−0.4471	−0.2852	−0.4106	31	20	18	0.0062
N	−0.1206	−0.3566	−0.7018	31	22	28	0.0035
NC	−0.5968	−0.8148	−0.6733	19	13	21	0.0044
C2	−0.1557	−0.3257	−0.2129	16	7	6	0.0111
N(	−0.3278	−0.0950	−0.4204	16	15	20	0.0027
N=	−0.7064	−1.2648	−0.2549	8	7	6	0.0033
N1	−1.7236	−1.9358	−0.7488	7	3	6	0.0079
cN	−0.1443	−1.5157	−1.8619	6	2	5	0.0098
4	−0.6547	−0.5008	−0.2820	4	4	1	0.0107
5(	−0.3569	−0.1286	−0.9625	2	1	0	1.0000
N2	−0.5617	−0.9860	−0.3725	2	1	0	1.0000

* NA, NP, and NC are frequencies of S_k_ or SS_k_ in the active training set, passive training set, and calibration set, respectively. The 2D interpretation of Sk and SSk is available in the literature [28].

**Table 3:** Comparison of the statistical parameters of different models for acute fish toxicity.

Models of Fish Acute Toxicity from the Literature					Models of Fish Toxicity Obtained by CORAL Software			
N_train_ *	D_train_	N_valid_	D_valid_	Method	N_train_	D_train_	N_valid_	D_valid_
211	-	14	0.97	LR [20]	158 ± 1	0.577 ± 0.10	53 ± 1	0.815 ± 0.07
86	0.67	25	0.83	PLS [49]	84 ± 3	0.711 ± 0.06	28 ± 1	0.947 ± 0.02
39	0.80	16	0.84	PCA [27]	42 ± 1	0.650 ± 0.09	14 ± 1	0.749 ± 0.05
					188 ± 2	0.457 ± 009	60 ± 2	0.673 ± 0.03

* N_train_ = the number of compounds in training set; N_valid_ = the number of compounds in validation set; D_train_ = determination coefficient for validation set; D_valid_ = determination coefficient for validation set; LR = linear regression; PLS = partial least square; and PCA = principial components analysis.

## Data Availability

The data are available in this article or its Supplementary Materials.

## Supplementary Materials

The following supporting information can be downloaded at https://www.mdpi.com/article/10.3390/ijms26199348/s1.

## Abbreviations

The following abbreviations are used in this manuscript:

QSAR	Quantitative structure–activity relationships
DCW	Descriptor of correlation weights
SMILES	Simplified Molecular Input Line Entry System
CCC	Concordance correlation coefficient
R^2^	Correlation coefficient
Q^2^	Leave-one-out cross-validated R^2^
RMSE	Root means squared error
MAE	Mean absolute error
F	Fischer F-ratio
TF	Target function
IIC	Index of ideality of correlation

## References

[1] Toropov A.A., Toropova A.P., Benfenati E. (2009). Additive SMILES-based carcinogenicity models: Probabilistic principles in the search for robust predictions. Int. J. Mol. Sci..

[2] Selvestrel G., Lavado G.J., Toropova A.P., Toropov A.A., Gadaleta D., Marzo M., Baderna D., Benfenati E. (2022). Monte Carlo models for sub-chronic repeated-dose toxicity: Systemic and organ-specific toxicity. Int. J. Mol. Sci..

[3] Toropova A.P., Toropov A.A., Fjodorova N. (2023). In silico simulation of impacts of metal nano-oxides on cell viability in THP-1 cells based on the correlation weights of the fragments of molecular structures and codes of experimental conditions represented by means of quasi-SMILES. Int. J. Mol. Sci..

[4] Jung U., Lee B., Kim G., Shin H.K., Kim K.-T. (2021). Nano-QTTR development for interspecies aquatic toxicity of silver nanoparticles between daphnia and fish. Chemosphere.

[5] Li Z., Lu T., Li M., Mortimer M., Guo L.-H. (2023). Direct and gut microbiota-mediated toxicities of environmental antibiotics to fish and aquatic invertebrates. Chemosphere.

[6] Mlnaříková M., Pípal M., Bláhová L., Bláha L. (2024). Is environmental risk assessment possible with the alternatives to acute fish toxicity test? Case study with pharmaceuticals. Environ. Sci. Eur..

[7] Pandey S.K., Ojha P.K., Roy K. (2020). Exploring QSAR models for assessment of acute fish toxicity of environmental transformation products of pesticides (ETPPs). Chemosphere.

[8] Wang S., Yan L.C., Zheng S.S., Li T.T., Fan L.Y., Huang T., Li C., Zhao Y.H. (2019). Toxicity of some prevalent organic chemicals to tadpoles and comparison with toxicity to fish based on mode of toxic action. Ecotoxicol. Environ. Saf..

[9] Furuhama A., Hayashi T.I., Yamamoto H. (2019). Development of QSAAR and QAAR models for predicting fish early-life stage toxicity with a focus on industrial chemicals. SAR QSAR Environ. Res..

[10] Sheffield T.Y., Judson R.S. (2019). Ensemble QSAR modeling to predict multispecies fish toxicity lethal concentrations and points of departure. Environ. Sci. Technol..

[11] Teixidó E., Leuthold D., de Crozé N., Léonard M., Scholz S. (2020). Comparative assessment of the sensitivity of fish early-life stage, Daphnia, and algae tests to the chronic ecotoxicity of xenobiotics: Perspectives for alternatives to animal testing. Environ. Toxicol. Chem..

[12] Lunghini F., Marcou G., Azam P., Enrici M.H., Van Miert E., Varnek A. (2020). Consensus QSAR models estimating acute toxicity to aquatic organisms from different trophic levels: Algae, Daphnia and fish. SAR QSAR Environ. Res..

[13] Yu X., Zeng Q. (2022). Random forest algorithm-based classification model of pesticide aquatic toxicity to fishes. Aquat. Toxicol..

[14] Drgan V., Vračko M., Roy K. (2021). Counter-propagation neural networks for modeling and read across in aquatic (fish) toxicity. Chemometrics and Cheminformatics in Aquatic Toxicology.

[15] Yu X. (2021). Support vector machine-based model for toxicity of organic compounds against fish. Regul. Toxicol. Pharmacol..

[16] Zhou L., Fan D., Yin W., Gu W., Wang Z., Liu J., Xu Y., Shi L., Liu M., Ji G. (2021). Comparison of seven in silico tools for evaluating of daphnia and fish acute toxicity: Case study on Chinese Priority Controlled Chemicals and new chemicals. BMC Bioinform..

[17] Meador J.P. (2021). The fish early-life stage sublethal toxicity syndrome—A high-dose baseline toxicity response. Environ. Pollut..

[18] Gu W., Li X., Du M., Ren Z., Li Q., Li Y. (2021). Identification and regulation of ecotoxicity of polychlorinated naphthalenes to aquatic food Chain (green algae-*Daphnia magna*-fish). Aquat. Toxicol..

[19] Pulido-Reyes G., Moreno-Martín G., Gómez-Gómez B., Navas J.M., Madrid Y., Fernández-Cruz M.L. (2024). Fish acute toxicity of nine nanomaterials: Need of pre-tests to ensure comparability and reuse of data. Environ. Res..

[20] Klüver N., Vogs C., Altenburger R., Escher B.I., Scholz S. (2016). Development of a general baseline toxicity QSAR model for the fish embryo acute toxicity test. Chemosphere.

[21] Austin T., Denoyelle M., Chaudry A., Stradling S., Eadsforth C. (2015). European chemicals agency dossier submissions as an experimental data source: Refinement of a fish toxicity model for predicting acute LC50 values. Environ. Toxicol. Chem..

[22] Roy K., Kar S. (2016). In silico models for ecotoxicity of pharmaceuticals. Methods Mol. Biol..

[23] Kidd K.A., Blanchfield P.J., Mills K.H., Palace V.P., Evans R.E., Lazorchak J.M., Flick R.W. (2007). Collapse of a fish population after exposure to a synthetic estrogen. Proc. Natl. Acad. Sci. USA.

[24] Tang J., Liu A., Chen K., Shi Y., Qiu X. (2025). Exposure to amitriptyline disturbs behaviors in adult zebrafish and their offspring via altering neurotransmitter levels. Comp. Biochem. Physiol. C Pharmacol..

[25] McCallum E.S., Krutzelmann E., Brodin T., Fick J., Sundelin A., Balshine S. (2017). Exposure to wastewater effluent affects fish behaviour and tissue-specific uptake of pharmaceuticals. Sci. Total Environ..

[26] European Medicines Agency (EMEA) (2024). Guideline on the Environmental Risk Assessment of Medicinal Products for Human Use—Revision 1.

[27] Sangion A., Gramatica P. (2016). Hazard of pharmaceuticals for aquatic environment: Prioritization by structural approaches and prediction of ecotoxicity. Environ. Int..

[28] Weininger D. (1988). SMILES, a Chemical Language and Information System: 1: Introduction to Methodology and Encoding Rules. J. Chem. Inf. Comput. Sci..

[29] Toropov A.A., Benfenati E. (2007). Optimisation of correlation weights of SMILES invariants for modelling oral quail toxicity. Eur. J. Med. Chem..

[30] Toropov A.A., Toropova A.P., Martyanov S.E., Benfenati E., Gini G., Leszczynska D., Leszczynski J. (2012). CORAL: Predictions of rate constants of hydroxyl radical reaction using representation of the molecular structure obtained by combination of SMILES and Graph approaches. Chemometr. Intell. Lab. Syst..

[31] Fatemi M.H., Malekzadeh H. (2015). CORAL: Predictions of retention indices of volatiles in cooking rice using representation of the molecular structure obtained by combination of SMILES and graph approaches. J. Iran. Chem. Soc..

[32] Kumar P., Kumar A. (2020). CORAL: QSAR models of CB1 cannabinoid receptor inhibitors based on local and global SMILES attributes with the index of ideality of correlation and the correlation contradiction index. Chemometr. Intell. Lab. Syst..

[33] Chopdar K.S., Dash G.C., Mohapatra P.K., Nayak B., Raval M.K. (2022). Monte-Carlo method-based QSAR model to discover phytochemical urease inhibitors using SMILES and GRAPH descriptors. J. Biomol. Struct. Dyn..

[34] Tabti K., Elmchichi L., Sbai A., Maghat H., Bouachrine M., Lakhlifi T. (2022). Molecular modelling of antiproliferative inhibitors based on SMILES descriptors using Monte-Carlo method, docking, MD simulations and ADME/Tox studies. Mol. Simul..

[35] Rezaie-keikhaie N., Shiri F., Ahmadi S., Salahinejad M. (2023). QSTR based on Monte Carlo approach using SMILES and graph features for toxicity toward Tetrahymena pyriformis. J. Iran. Chem. Soc..

[36] Ouabane M., Zaki K., Tabti K., Alaqarbeh M., Sbai A., Sekkate C., Bouachrine M., Lakhlifi T. (2024). Molecular toxicity of nitrobenzene derivatives to tetrahymena pyriformis based on SMILES descriptors using Monte Carlo, docking, and MD simulations. Comput. Biol. Med..

[37] Toropova A.P., Toropov A.A. (2017). The index of ideality of correlation: A criterion of predictability of QSAR models for skin permeability?. Sci. Total Environ..

[38] Toropova A.P., Toropov A.A., Viganò E.L., Colombo E., Roncaglioni A., Benfenati E. (2022). Carcinogenicity prediction using the index of ideality of correlation. SAR QSAR Environ. Res..

[39] Toropov A.A., Toropova A.P., Roncaglioni A., Benfenati E. (2023). In silico prediction of the mutagenicity of nitroaromatic compounds using correlation weights of fragments of local symmetry. Mutat. Res. Genet. Toxicol. Environ. Mutagen..

[40] Javidfar M., Ahmadi S. (2020). QSAR modelling of larvicidal phytocompounds against Aedes aegypti using index of ideality of correlation. SAR QSAR Environ. Res..

[41] Ghiasi T., Ahmadi S., Ahmadi E., Talei Bavil Olyai M.R., Khodadadi Z. (2021). The index of ideality of correlation: QSAR studies of hepatitis C virus NS3/4A protease inhibitors using SMILES descriptors. SAR QSAR Environ. Res..

[42] Ahmadi S., Lotfi S., Kumar P. (2022). Quantitative structure–toxicity relationship models for predication of toxicity of ionic liquids toward leukemia rat cell line IPC-81 based on index of ideality of correlation. Toxicol. Mech. Methods.

[43] Duhan M., Sindhu J., Kumar P., Devi M., Singh R., Kumar R., Lal S., Kumar A., Kumar S., Hussain K. (2022). Quantitative structure activity relationship studies of novel hydrazone derivatives as α-amylase inhibitors with index of ideality of correlation. J. Biomol. Struct. Dyn..

[44] Kumar A., Kumar P., Singh D. (2022). QSRR modelling for the investigation of gas chromatography retention indices of flavour and fragrance compounds on Carbowax 20 M glass capillary column with the index of ideality of correlation and the consensus modelling. Chemometr. Intell. Lab. Syst..

[45] Goyal S., Rani P., Chahar M., Hussain K., Kumar P., Sindhu J. (2023). Quantitative structure activity relationship studies of androgen receptor binding affinity of endocrine disruptor chemicals with index of ideality of correlation, their molecular docking, molecular dynamics and ADME studies. J. Biomol. Struct. Dyn..

[46] Bhawna, Kumar S., Kumar P., Kumar A. (2024). Correlation intensity index-index of ideality of correlation: A hyphenated target function for furtherance of MAO-B inhibitory activity assessment. Comput. Biol. Chem..

[47] Khan K., Baderna D., Cappelli C., Toma C., Lombardo A., Roy K., Benfenati E. (2019). Ecotoxicological QSAR modeling of organic compounds against fish: Application of fragment based descriptors in feature analysis. Aquat. Toxicol..

[48] Petoumenou M.I., Pizzo F., Cester J., Fernández A., Benfenati E. (2015). Comparison between bioconcentration factor (BCF) data provided by industry to the European Chemicals Agency (ECHA) and data derived from QSAR models. Environ. Res..

[49] de Morais E Silva L., Alves M.F., Scotti L., Lopes W.S., Scotti M.T. (2018). Predictive ecotoxicity of MoA 1 of organic chemicals using in silico approaches. Ecotoxicol. Environ. Saf..

[50] Toropova A.P., Toropov A.A., Benfenati E., Gini G. (2011). Co-evolutions of correlations for QSAR of toxicity of organometallic and inorganic substances: An unexpected good prediction based on a model that seems untrustworthy. Chemometr. Intell. Lab. Syst..

[51] Vukomanović P., Stefanović M., Stevanović J.M., Petrić A., Trenkić M., Andrejević L., Lazarević M., Sokolović D., Veselinović A.M. (2024). Monte Carlo optimization method based QSAR modeling of placental barrier permeability. Pharm. Res..

[52] Šarić S., Kostić T., Lović M., Aleksić I., Hristov D., Šarac M., Veselinović A.M. (2024). In silico development of novel angiotensin-converting-enzyme-I inhibitors by Monte Carlo optimization based QSAR modeling, molecular docking studies and ADMET predictions. Comput. Biol. Chem..

[53] Nikolić N., Kostić T., Golubović M., Nikolić T., Marinković M., Perić V., Mladenović S., Veselinovic A. (2023). Monte Carlo optimization based QSAR modeling of angiotensin II receptor antagonists. Acta Chim. Slov..

[54] Toropova A.P., Raškova M., Raška I., Toropov A.A. (2021). The sequence of amino acids as the basis for the model of biological activity of peptides. Theor. Chem. Acc..

[55] Ciemny M.P., Badaczewska-Dawid A.E., Pikuzinska M., Kolinski A., Kmiecik S. (2019). Modeling of disordered protein structures using Monte Carlo simulations and knowledge-based statistical force fields. Int. J. Mol. Sci..

[56] Especial J.N.C., Rey A., Faísca P.F.N. (2022). A note on the effects of linear topology preservation in Monte Carlo simulations of knotted proteins. Int. J. Mol. Sci..

[57] Penabeï S., Meesungnoen J., Jay-Gerin J.-P. (2024). Comparative analysis of cystamine and cysteamine as radioprotectors and antioxidants: Insights from Monte Carlo chemical modeling under high linear energy transfer radiation and high dose rates. Int. J. Mol. Sci..

[58] Wüstner D., Sklenar H. (2014). Atomistic Monte Carlo simulation of lipid membranes. Int. J. Mol. Sci..

[59] Peukert D., Kempson I., Douglass M., Bezak E. (2019). Gold nanoparticle enhanced proton therapy: Monte Carlo modeling of reactive species’ distributions around a gold nanoparticle and the effects of nanoparticle proximity and clustering. Int. J. Mol. Sci..

[60] Li S.W., Lin A.Y.C. (2015). Increased acute toxicity to fish caused by pharmaceuticals in hospital effluents in a pharmaceutical mixture and after solar irradiation. Chemosphere.

[61] Yang J.-S., Panchangam S.C., Lin A.Y.C. (2025). Exploring simulated sunlight sulfite process for enhanced removal of mixed pharmaceutical and personal care products from aqueous solution. J. Water Process Eng..

[62] Coors A., Ross Brown A., Maynard S.K., Nimrod Perkins A., Owen S., Tyler C.R. (2023). Minimizing Experimental Testing on Fish for Legacy Pharmaceuticals. Environ. Sci. Technol..

[63] Khan K., Benfenati E., Roy K. (2019). Consensus QSAR modeling of toxicity of pharmaceuticals to different aquatic organisms: Ranking and prioritization of the DrugBank database compounds. Ecotoxicol. Environ. Saf..

[64] Khan K., Khan P.M., Lavado G., Valsecchi C., Pasqualini J., Baderna D., Marzo M., Lombardo A., Roy K., Benfenati E. (2019). QSAR modeling of Daphnia magna and fish toxicities of biocides using 2D descriptors. Chemosphere.

[65] Kar S., Roy K. (2010). First report on interspecies quantitative correlation of ecotoxicity of pharmaceuticals. Chemosphere.

